# t(1;4) translocation in a child with acute lymphoblastic leukemia: a case report

**DOI:** 10.1186/s13256-023-04270-9

**Published:** 2023-12-12

**Authors:** Chaimae El Mahdaoui, Nezha Hda, Bouchra Oukkache, Hind Dehbi, Nisrine Khoubila, Abdellah Madani, Siham Cherkaoui

**Affiliations:** 1 Laboratory of Cellular and Molecular Pathology, Faculty of Medicine and Pharmacy, Hassan II University of Casablanca, Casablanca, Morocco; 2 Hda Laboratories of Medical Biology Analysis, Casablanca, Morocco; 3 Hematology Laboratory, Ibn Rochd University Hospital, Casablanca, Morocco; 4 Laboratory of Medical Genetics, Ibn Rochd University Hospital, Casablanca, Morocco; 5 Hematology and Pediatric Oncology Department of August 20 Hospital, Ibn Rochd University Hospital, Casablanca, Morocco

**Keywords:** Acute lymphoblastic leukemia, Cytogenetics, B-ALL, Case report

## Abstract

**Background:**

Acute lymphoblastic leukemia is the most common childhood cancer, with an 80% frequency in children between 1 and 10 years old. The outcome and prognosis of acute lymphoblastic leukemia in children depends on various factors, such as age, clinical and biological features, and cytogenetic factors.

**Case presentation:**

We report the case of a pediatric patient, a 4-year-old Moroccan female who was referred to the Hematology and Oncology Department of 20 August 1953 Hospital in Casablanca and diagnosed with B-cell acute lymphoblastic leukemia associated with a rare genetic chromosomal abnormality.

**Conclusion:**

Translocation (1;4)(p21;p15) is a relatively rare chromosomal abnormality found in human leukemia and was never described isolated in pediatric B-cell acute lymphoblastic leukemia patients. It showed a good evolution by complete remission and recovery of this patient after receiving all chemotherapy and after 8 years of follow-up.

## Background

Acute lymphoblastic leukemia (ALL) is a malignant neoplasm characterized by the uncontrolled proliferation of lymphoid progenitor cells. Cytogenetic abnormalities play a crucial role in the prognosis and treatment strategies for ALL patients. While several well-known chromosomal aberrations have been extensively studied and documented in ALL, rare translocations continue to emerge, presenting unique challenges in diagnosis, risk stratification, and therapeutic decision-making [1, 2].

ALL commonly arises from a series of genetic alterations and, in most ALL subtypes, the interaction of these alterations.

ALL commonly arises from a series of genetic alterations and, in most ALL subtypes, the interaction of these alterations.

In B-cell acute lymphoblastic leukemia (B-ALL), common translocations and number chromosomal abnormalities in children include t(12;21) [ETV6-RUNX1] with a rate of 25%, t(1;19) [TCF3-PBX1] with 5%, t(9;22) [BCR-ABL1] in 3% of ALL, and translocations involving the *MLL* gene with various partner fusion genes (5%). Gains in whole chromosomes, or high hyperdiploidy (> 50 chromosomes), account for 25% of childhood ALL, whereas hypodiploidy (< 44 chromosomes) accounts for ~1% of cases [3].

Herein, we present a compelling case of a pediatric patient diagnosed with ALL containing a rare translocation t(1;4). Translocations involving the chromosomal breakpoints 1q and 4p are infrequently reported in the literature, making this case noteworthy for its rarity and potential implications in the management of pediatric ALL [4].

The translocation t(1;4) involves the reciprocal exchange of genetic material between the long arm of chromosome 1 and the short arm of chromosome 4, leading to a fusion of genes or gene disruption. In recent years, advancements in molecular techniques, such as fluorescence *in situ* hybridization (FISH) and polymerase chain reaction (PCR), have enhanced our ability to detect and characterize such genetic abnormalities accurately [5].

This case report aims to contribute to the growing body of knowledge on rare genetic alterations in ALL, shedding light on the clinical, molecular, and prognostic significance of the t(1;4) translocation.

## Case presentation

### General information and physical examination findings

We present a case of a 4-year-old Moroccan girl who was diagnosed with B-ALL associated with a t(1;4) chromosomal abnormality in 2016. Her father has laryngeal cancer. She has no medical history nor past interventions.

She was admitted 20 days after showing signs of pharyngitis and asthenia of increasing intensity associated with paleness and fever.

### Clinical findings

The clinical examination showed an abscess fistulated in the left labia majora.

Her chest X-ray showed some parenchymal infiltrations with micronodules. Other than that, her echo heart and ultrasound were normal.

### Diagnostic assessment

The diagnosis of ALL is based on clinical and biological features and the morphological immunophenotyping identification of leukemic bone marrow blasts. Genetic alterations are very important to determine the treatment and prognosis of the patient.

## Morphology

Based on clinical observation and hemogram results, the patient underwent bone marrow aspiration to study and identify bone marrow cells.

Her initial cell blood count (CBC) revealed the following: hemoglobin (Hb): 6.00 g/dL; mean corpuscular volume (MCV): 84.1 fL; mean corpuscular hemoglobin concentration (MCHC): 35.3 g/dL, white blood cell (WBC) count: 8100 cells/mm^3^; neutrophil (Neu) count: 570.10^3^ cells/mm^3^, and platelet (PLT) count: 41,000 cells/mm^3^.

Thus, bone marrow aspiration from the 4-year-old patient showed rich bone marrow with 97% blast cells of heterogeneous size with very reduced cytoplasm, a nucleus with condensed chromatin, basophil cells without granulations, an absence of megakaryocytes, 2% myelocytes, and 2% lymphocytes. This specificity indicates ALL subtype 2.

## Immunophenotype

In our case, the immunophenotype was determined with flow cytometry on bone marrow and showed an aberrant expression of CD13, which is considered a myeloid marker (Table 1).

**Table 1:** Results of immunophenotyping of the patient

Marker	Results (%)
CD117	1.03
CD15	0.16
CD13	31.06
CD33	0.01
CD19	94.35
CD22c	92.61
CD20	2.04
CD10	98.14
CD3s	0.22
CD2	0.02
CD103	0.03
HLADR	88.81
PTPRC	90.38

## Conventional cytogenetics

The sample containing the blasts is cultured and treated to obtain a sufficient number of mitotic cells, which are then analyzed by conventional cytogenetics. The preferred sample sources are bone marrow or peripheral blood when they contain blast cells. The sample must be taken under sterile conditions in a heparinized tube.

In our study, G-banded analysis of 20 metaphases was performed using the standard cytogenetic protocol, and karyotypes were described according to the International System for Human Cytogenetic Nomenclature 2009 [6].

## Fluorescence *in situ* hybridization (FISH)

Fluorescence *in situ* hybridization (FISH) was performed on 200 mitotic cells following the manufacturer’s guidelines (Abbott Molecular, Inc., Des Plaines, IL, USA). The probe ISI double-labeling DNA probes, break-apart specific locus *MLL* 11g23 (Vysis) status, and whole chromosome paint probes (WCP) for chromosomes 1, 6, and 11 were used to confirm the karyotype results.

The results of our case showed the absence of rearrangement and/or deletion of the *MLL* gene in all observed cells (200 mitoses).

nuc ish(5’*MLL*, 3’*MLL*, 5’*MLL* con 3’*MLL*) × 2 [200].

46, xx ISH 5’*MLL*,3’*MLL* 5’*MLL*con 3’*MLL*) × 2 [16].

Her karyotype was made on 20 mitoses and unveiled a clone with a translocation between the short arm of chromosome 1 and the short arm of chromosome 4 in 10 mitoses, 46,XX, t(1;4)(p21;p15)[10]/46,XX[10], and complementary FISH was performed.

**Fig. 1:**
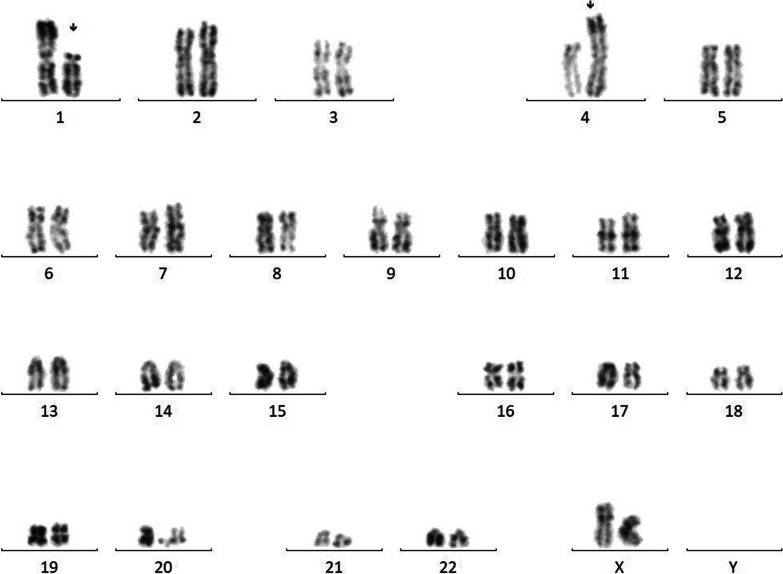
Image of the patient’s karyotype showing t(1;4)

Karyotype images for our patient were collected from the laboratory (Fig. 1).

The patient had no challenges accessing the diagnostic testing, as the medication was available and accessible in the hospital.

### Therapeutic intervention and follow-up

After antibiotics and symptomatic treatment, she started a standard risk induction regimen in November 2016 and was in incomplete remission. Then, she underwent chemotherapy according to the FRALLE 2000-B2 protocol.

The patient followed her treatment correctly. She finished her treatment after 2 years of maintenance chemotherapy in September 2019.

The patient was seen after the end of her treatment; she is now 11 years old and in complete remission.

## Discussion

Through this case report, we strived to provide a comprehensive description of the clinical presentation, laboratory findings, and treatment outcomes in a pediatric patient with t(1;4)(p21;p15) ALL. Additionally, we aimed to discuss the potential implications of this translocation on disease progression, response to therapy, and long-term prognosis.

The diagnosis of ALL is established by identifying more than 20% blast cells in all hematopoietic cells [7]. This classification is primarily based on the microscopic appearance of the leukemic cells observed in May–Grünwald Giemsa-stained smears.

Immunophenotyping is an essential part of the diagnosis and classification of ALL to identify the lymphoid nature of the blasts. Unlike morphologic features, the lineage of ALL established through immunophenotyping subdivides the disease into two broad, clinically and biologically meaningful categories: precursor B-cell ALL (B-ALL) and precursor T-cell ALL (T-ALL) [8]. This analysis also specifies the stage of maturation of the lymphoblasts according to the classification of the European Group for the Immunological Characterization of Leukemia (EGIL).

Most cases of B-ALL show expression of one or several myeloid-associated antigens, most often CD13 and CD33, and less often CD11b, CD15, and CD66c. The pattern of immunophenotypic aberrations correlates to some extent with the underlying genetic lesion in B-ALL [9].

The mixed-lineage leukemia (*MLL*) gene, situated on chromosome 11q23, plays a significant role in chromosomal translocations found in a specific subtype of acute leukemia. This subtype accounts for approximately 10% of acute lymphoblastic leukemia cases and 2.8% of acute myeloid leukemia cases [10].

This report underscores the importance of meticulous cytogenetic analysis and molecular characterization in pediatric ALL cases, emphasizing the need for further research to elucidate the biological mechanisms underlying rare translocations such as t(1;4) [11].

​The translocation (1;4) (p21;p15) is a relatively rare chromosomal abnormality found in human leukemia pediatric ALL. It was not found isolated in the chromosomal database associated with B-ALL [12] nor in the atlas genetic database (Cf.Atlas genetics oncology).

In a study in Turkey, the translocation (1;4)(q42;q22) was found in two pediatric patients with acute myeloid leukemia [13].

This abnormality was also reported in an elderly patient as 46,XY,t(1;4)(p10;q10) in the Edouard Herriot Hospital Experience [14] and in a young adult of 22 years old with T-ALL in Germany with a karyotype described as following 46,XY,t(1;4)(p22;q27) [15].

Another study showed a new translocation t(1;11;4) in the peripheral blood of a patient but with congenital acute myeloblastic leukemia (AML) [16].

Virginie Eclache A *et al*. described a case of a 7-month-old patient with acute lymphoblastic leukemia involving four chromosomes, that is, 1, 4, 6, and 11, resulting in t(1;4;6;11)(q31;q27;q22;q23), and fluorescent *in situ* hybridization (FISH) confirmed mixed-lineage leukemia (*MLL*) gene rearrangement [17].

This translocation was also reported in a case of adult chronic lymphocytic leukemia in France in 2002 but as a constitutional translocation t(1;4)(p21;p15) [18].

By reporting this case, we contribute to the literature and emphasize the need for increased awareness of uncommon cytogenetic abnormalities in pediatric ALL. It is our hope that this report will facilitate future research endeavors, ultimately leading to improved risk stratification, targeted therapeutic interventions, and better clinical outcomes for patients with ALL and similar genetic alterations.

## Conclusion

In conclusion, the present case report highlights a rare instance of t(1;4) translocation in a patient with B-ALL who showed a good evolution after 8 years of follow-up.

## Data Availability

The datasets used and/or analyzed during the current study are available from the corresponding author on reasonable request.

## Abbreviations

ALL: Acute lymphoblastic leukemia
B-ALL: B-cell acute lymphoblastic leukemia
ETV6: ETS variant transcription factor 6
RUNX1: Runt-related transcription factor 1
PBX1: Pre-B-cell leukemia homeobox 1
TCF3: Transcription factor 3
BCR: Breakpoint cluster region
ABL: Abelson
MLL: Mixed lineage leukemia gene
FISH: Fluorescence *in situ* hybridization
PCR: Polymerase chain reaction
CBC: Cell blood count
Hb: Hemoglobin
MCV: Mean corpuscular volume
MCHC: Mean corpuscular hemoglobin concentration
WBC: White blood cell
Neu: Neutrophil
PLT: Platelets
T-ALL: T-cell acute lymphoblastic leukemia
EGIL: European Group for the Immunological Characterization of Leukemia
CD: Cluster of differentiation antigen
DNA: Deoxyribonucleic acid
WCP: Whole chromosome paint probe
